# Salinity, not genetic incompatibilities, limits the establishment of the invasive hybrid cattail *Typha* × *glauca* in coastal wetlands

**DOI:** 10.1002/ece3.6831

**Published:** 2020-10-01

**Authors:** Kathryn Tisshaw, Joanna Freeland, Marcel Dorken

**Affiliations:** ^1^ Environmental and Life Sciences Graduate Program Trent University Peterborough ON Canada; ^2^ Department of Biology Trent University Peterborough ON Canada

**Keywords:** bioinvasions, cattails, fitness, hybrid zones, local adaptation, salinity, *Typha*

## Abstract

Hybrids of a single pair of parent species can be much more common in some geographical regions than in others. The reasons for this are not well understood, but could help explain processes such as species diversification or the range expansion of invasive hybrids. The widespread cattails *Typha latifolia* and *T. angustifolia* seldom hybridize in some parts of their range, but in other areas produce the dominant hybrid *T*. × *glauca*. We used a combination of field and greenhouse experiments to investigate why *T*. × *glauca* has invaded wetlands in the Laurentian Great Lakes region of southern Ontario, Canada, but is much less common in the coastal wetlands of Nova Scotia (NS) in eastern Canada. One potentially important environmental difference between these two regions is salinity. We therefore tested three hypotheses: (1) *T. latifolia* and *T. angustifolia* in NS are genetically incompatible; (2) the germination or growth of *T*. × *glauca* is reduced by salinity; and (3) *T. latifolia,* a main competitor of *T*. × *glauca*, is locally adapted to saline conditions in NS. Our experiments showed that NS *T. latifolia* and *T. angustifolia* are genetically compatible, and that saline conditions do not impede growth of hybrid plants. However, we also found that under conditions of high salinity, germination rates of hybrid seeds were substantially lower than those of NS *T. latifolia*. In addition, germination rates of NS *T. latifolia* were higher than those of Ontario *T. latifolia,* suggesting local adaptation to salinity in coastal wetlands. This study adds to the growing body of literature which identifies the important roles that local habitat and adaptation can play in the distributions and characteristics of hybrid zones.

## Graphical abstract

The invasive cattail hybrid *Typha* × *glauca* dominates wetlands around the Laurentian Great Lakes. We investigated why it is much less common in the maritime provinces of Canada and found that salinity inhibits seed germination and hence the establishment of hybrids in coastal wetlands.

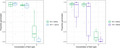

## 1 INTRODUCTION

Hybridization is widespread in plants, occurring in an estimated 40% of vascular plant families, and has played an important role in species diversification (Ellstrand et al., 1996; Mallet, 2005). However, interspecific hybrids are more common in some taxonomic groups than others (Rieseberg et al., 2006; Whitney et al., 2010), and their frequencies (reflecting the abundance of hybrids) can also vary considerably between different contact zones (regions of sympatry) of a single pair of parental species (e.g., Aldridge & Campbell, 2009; Li & Maki, 2015; Watano et al., 2004). In contact zones with high frequencies of hybrids, most individuals have characteristics intermediate to those of the parents; this represents unimodal character distribution and reflects weak barriers to hybridization (e.g., Burgess et al., 2005; Jiggins & Mallet, 2000). Under these conditions, species reproductive barriers may be eroded, potentially leading to a hybrid swarm. Conversely, both parental types are maintained in contact zones where hybrids are rare, reflecting strong barriers to hybridization and bimodal distributions in which the phenotypes of both parental species are maintained (Harrison & Bogdanowicz, 1997). When hybridization barriers are of varying strength at different contact zones,, a pair of species may form either unimodal or bimodal distributions across their sympatric geographical distribution (Albaladejo & Aparicio, 2007; Aldridge & Campbell, 2009; Barton & Hewitt, 2003; Oberprieler et al., 2011; Watano et al., 2004; Zeng et al., 2011).

Understanding why the strength of reproductive isolation varies across contact zones for a single species pair can give us insight into a range of evolutionary and applied questions, for example, with respect to speciation or the spread of novel invasive hybrids. Past explanations for this variation include intrinsic genetic forces that manifest as local differences in genetic incompatibilities between the progenitor species. (Buerkle & Rieseberg, 2001), or ecological and environmental conditions across contact zones that affect either the formation or the survival of hybrids (Aldridge & Campbell, 2009; Bleeker & Hurka, 2001; Howard, 2001; Jiggins & Mallet, 2000; Li & Maki, 2015; Watano et al., 2004). Ultimately, the interaction of environments and genotypes should determine whether hybrid fitness will be higher, lower, or comparable to the fitness of parents across different contact zones (Campbell et al., 2008; Taylor et al., 2009); however, there have been relatively few investigations of this phenomenon and thus generalizations are lacking.

The widespread wetland macrophyte genus *Typha* includes multiple pairs of species that are capable of hybridization (e.g., Bansal et al., 2019; Ciotir et al., 2017; Grace & Harrison, 1986; Kirk et al., 2011). Two species that have been particularly well‐studied are *T. latifolia* (broadleaf cattail) and *T. angustifolia* (narrowleaf cattail), which sometimes hybridize to form *T*. × *glauca* (Smith, 1967). However, the frequencies of *T*. × *glauca* differ dramatically throughout the sympatric range of the parent species. At one extreme, no hybrids were found in large areas of China where *T. latifolia* and *T. angustifolia* co‐occur (Zhou et al., 2016), and only low levels of hybridization have been reported from Europe (Ciotir et al., 2017; Nowińska et al., 2014). Similarly, hybrids occur at low frequencies in some geographic regions of North America, including the maritime provinces of Canada where the contact zones remain largely bimodal (Freeland et al., 2013; Kirk et al., 2011; Pieper et al., 2020). However, in other regions of North America, most notably those surrounding the Laurentian Great Lakes and St. Lawrence Seaway, contact zones are often unimodal; here, *T*. × *glauca* is an invasive taxon that dominates wetlands and is often more abundant than its parental species (Freeland et al., 2013; Kirk et al., 2011; Travis et al., 2010). Additionally, hybrids in this region are fertile and capable of forming advanced‐generation and back‐crossed hybrids (Pieper et al., 2017). In North America, *T. latifolia* is native (Grace & Harrison, 1986), whereas *T. angustifolia* is introduced, albeit several centuries ago (most likely around the time of early European colonization; Ciotir & Freeland, 2016; Ciotir et al., 2013). Because wetlands that are dominated by *Typha × glauca* suffer from reduced native plant diversity (Larkin et al., 2012; Tuchman et al., 2009; Vaccaro et al., 2009), reduced macroinvertebrate abundance (Lawrence et al., 2016), and altered sediment nutrient pools and microbial communities (Geddes et al., 2014; Lishawa et al., 2014), there is a particular need to better understand why these hybrids are so successful in some areas but not in others.

The successful biological invasion by *T*. × *glauca* in some regions of North America can be partially attributed to greater clonal growth, height, and seed germination compared to its progenitor species (Bunbury‐Blanchette et al., 2015; Szabo et al., 2018; Travis et al., 2011; Zapfe & Freeland, 2015). However, the paucity of hybrids in other areas where *T. latifolia* and *T. angustifolia* co‐exist may be explained by local environmental conditions. Water and soil salinity in wetlands is one potentially important environmental difference between the inland Great Lakes Region of Ontario (ON) where *T*. × *glauca* flourishes, and the maritime province of Nova Scotia (NS) where *T*. × *glauca* is less common. Competitive species interactions can also influence the distributions of taxa (Bertness, 1991; Case et al., 2005), and *T. latifolia*, which is the dominant *Typha* species in NS, may be a more effective competitor against *T*. × *glauca* in that region compared to regions around the Great Lakes. In this study, we therefore used a combination of field and common garden experiments to test three hypotheses that could explain low hybrid frequency in a contact zone of *T. latifolia* and *T. angustifolia* in eastern Canada: (1) inherent genetic incompatibilities between *T. latifolia* and *T. angustifolia* in NS lead to inviable hybrids; (2) environmental salinity impedes the germination or growth of *T*. × *glauca*; and (3) NS *T. latifolia* is locally adapted to saline conditions. We first generated field crosses between *T. angustifolia* and *T. latifolia* in NS and quantified seed set and germination rates to test the hypothesis that hybrids are inviable. We then used common garden experiments to grow plants under controlled conditions (including a range of known salinity levels) in order to compare germination and growth rates of NS *T*. × *glauca*, NS *T. latifolia*, and ON *T. latifolia*. This approach allowed us to assess the interaction between genes and environment and hence test the hypotheses that high salinity reduces the fitness of hybrids and *T. latifolia* from freshwater habitats (ON), but not the fitness of *T. latifolia* from a maritime region (NS). Collectively, these experiments allow us to better understand the pronounced variation in *T*. × *glauca* frequencies across contact zones, which in turn could help us to predict whether hybrid cattails may become invasive in regions outside those surrounding the Laurentian Great Lakes and St. Lawrence Seaway.

## 2 METHODS

### 2.1 Genetic compatibility experiment

This experiment tested the hypothesis that interspecific hybridization between NS progenitors produces relatively few viable seeds. The experiment began with hand‐pollinations in NS, Canada. We collected pollen from 11 *T. latifolia* individuals spaced at least 5 m apart at Miner's Marsh (Table 1) from June 23 to July 11, 2017. Pollen was collected by placing a 24 cm × 28 cm glassine envelope over the dehiscent staminate spike, bending the stem, and shaking pollen into the envelope. Pollen type varies between the three taxa, with *T. latifolia* producing tetrad pollen, *T. angustifolia* producing monad pollen, and *T*. × *glauca* producing a mixture of pollen types, including tetrads and monads (Krattinger, 1975). We used a compound microscope at 40 × magnification to confirm that in each case we had only tetrad pollen grains and hence pollen from *T. latifolia* and not a hybrid. The envelopes were sealed with tape, inserted into Ziploc bags, and placed in the fridge for storage; *Typha* pollen maintains 90% viability when stored in these conditions for up to 50 days (Buitink et al., 1998).

**TABLE 1:** Sources of plants used in each of the three experiments: genetic compatibility, seed germination across different salinities, and plant growth in different salinities

Site name	Location	Role of each site		
		Plants used to create hybrids for genetic compatibility experiment	Source of seeds for experiment comparing germination in different salinities	Source of seeds for experiment comparing plant growth in different salinities
NS				
Miners Marsh	Kentville, NS (45.080, −64.490)	Source of pollen (*T. latifolia*) (11 plants)	Seeds from 4 *T. latifolia* plants	Seeds from 3 *T. latifolia* plants
Blueberry Run Trail (BR)	Chezzetcook, NS (44.739, −63.253)	Eight maternal plants (*T. angustifolia*)	Seeds from 2 *T. × glauca* plants	Seeds from 2 *T. × glauca* plants
Irishman's Road (IR)	Windsor, NS (44.975, −64.072)	Eight maternal plants (*T. angustifolia*)	Seeds from 4 *T. latifolia* plants, 2 *T. × glauca* plants	Seeds from 4 *T. latifolia* plants, 2 *T. × glauca* plants
Brooklyn Street (BS)	Kentville, NS (45.081, −64.501)	Eight maternal plants (*T. angustifolia*)	Seeds from 2 *T. × glauca* plants	Seeds from 2 *T. × glauca* plants
Exit 7 (E7)	Falmouth, NS (45.001, −64.157)	Eight maternal plants (*T. angustifolia*)	Seeds from 2 *T. × glauca* plants	Seeds from 2 *T. × glauca* plants
Exit 9 (E9)	Avonport, NS (45.100, −64.263)	Eight maternal plants (*T. angustifolia*)	Seeds from 2 *T. × glauca* plants	Seeds from 2 *T. × glauca* plants
Lawrencetown Coastal Heritage Park	44.645, −63.341 (Lawrencetown)		Seeds from 1 *T. latifolia* plant	
ON				
Elm Tree Road	44.272, −78.770 (Little Britain)		Seeds from 4 *T. latifolia* plants	Seeds from 3 *T. latifolia* plants
Sand Bar Road	44.236, −78.773 (Little Britain)		Seeds from 3 *T. latifolia* plants	Seeds from 2 *T. latifolia* plants
Cottage Road	44.230, −78.814 (Little Britain)		Seeds from 4 *T. latifolia* plants	Seeds from 2 *T. latifolia* plants
University and 4th Line	44.335, −78.282 (Peterborough)		Seeds from 3 *T. latifolia* plants	Seeds from 2 *T. latifolia* plants
Heron Landing Golf Course	44.267, −78.396 (Peterborough)		Seeds from 3 *T. latifolia* plants	Seeds from 2 *T. latifolia* plants

Hand‐pollinations of *T. angustifolia* pistillate spikes were done at five sites (Table 1). Maternal plants were provisionally identified to taxon based on morphology (Kirk et al., 2011; Snow et al., 2010), and later confirmed to taxon in the laboratory using microsatellite genotyping, following the methods of (Pieper et al., 2018). Each *T. angustifolia* plant was emasculated before either the male or the female inflorescence had emerged from the sheath, and the remaining pistillate spikes were covered with Canvasback^®^ pollination bags (Seedburo, Des Plaines, IL). Immediately prior to each hand pollination, we ensured that *T. angustifolia* individuals were receptive to pollen using the peroxidase enzyme test (Zeisler, 1938), in which excised pistils were submersed in a 3% hydrogen peroxide solution on a glass slide. Bubbling on the stigma, which we observed with a 10× hand lens, indicates the presence of peroxidase and hence stigma receptivity. Using a clean 1‐inch paintbrush for each individual, we brushed all sides of each inflorescence with pollen. All pollinations were conducted between June 30 and July 12, 2017. Once pollinated, we immediately re‐bagged the inflorescences to avoid further cross‐pollination. Bags were removed when all of the staminate spikes from all *Typha* plants within each site had stopped shedding pollen.

We collected fruits from September 11, 2017 to September 27, 2017 and transported them to Trent University, Ontario. Fruit storage and collection, and seed preparation for germination, followed the methods of (Ahee et al., 2015). We cleaned 0.50 g of fruit from each of ten randomly selected maternal plants of F1 hybrids and placed cleaned achenes (hereafter referred to as seeds) in individual Petri dishes with deionized water. Each Petri dish was photographed on a light table to record the total number of seeds and then moved to a greenhouse with a daily photoperiod of 12 hr and temperatures of approximately 18–20°C. Seeds were considered to have germinated once a radicle had emerged. After seven days, we counted the number of germinated seeds. An index of seed set was calculated from the total number of seeds per 0.50 g of fruit (after Ahee et al., 2015).

### 2.2 Environmental limitations of hybrid formation

The following common garden experiments investigate whether the response to salinity varies among populations and taxa, resulting in genetic × environment interactions. Specifically, we tested the hypotheses that (1) seed germination and seedling growth of *T*. × *glauca* is inhibited in saline conditions, and (2) NS *T. latifolia* is locally adapted to saline conditions.

#### 2.2.1 Germination in different salinities

Tidal marshes in NS, where *T. latifolia* dominates, exhibit strong seasonal variation in salinity concentrations. Average salinities range from 0 to 8 ppt depending on their exposure to regular tidal inundations, and maximum salinities (primarily from sodium and chloride ions) exceed 20 ppt (Bowron et al., 2013; Garbary et al., 2008; Porter et al., 2015). Salinity in southern ON near the Laurentian Great Lakes, where *T. × glauca* is common, is much lower: wetlands are freshwater, and although road de‐icing salts have contributed to pulses of higher chloride concentrations in the spring, these levels do not normally exceed 2 ppt (Wallace & Biastoch, 2016; Winter et al., 2011). Inland sites in NS are broadly intermediate, with salinity levels of 4 ppt reported from one of the sites included in this study (Miners Marsh) (Banks, 2013).

We conducted two experiments to compare the germination rates under different salinity conditions between (1) NS *T. latifolia* and NS *T*. × *glauca* to determine whether high salinity impedes germination of *T*. × *glauca* seeds more than *T. latifolia* seeds, and (2) NS *T. latifolia* and ON *T. latifolia* to investigate the possibility that NS *T. latifolia* is locally adapted to saline conditions. The NS *T*. × *glauca* seeds were generated by hand‐crosses in the previously described genetic compatibility experiment. The NS and ON *T. latifolia* seeds were collected in September and October 2017, respectively. As before, maternal plants were first provisionally identified in the field based on morphology, and later confirmed as *T. latifolia* from microsatellite genotypes. The *T. latifolia* seeds were collected from open‐pollinated plants. Asymmetric hybridization means that *T. latifolia* produces very few seeds when pollinated by *T. angustifolia* or *T*. × *glauca* (Pieper et al., 2017), and *T. latifolia* inflorescences are therefore very unlikely to contain *T*. × *glauca* seeds; nevertheless, we used the previously described microsatellite genotyping methods to confirm that a subset (three to nine seedlings from each *T. latifolia* inflorescence) of offspring were indeed *T. latifolia*. In total, the seeds for these two experiments came from nine *T. latifolia* inflorescences from three sites in NS, twelve *T. latifolia* inflorescences from five sites in Ontario, and 10 *T. *× *glauca* inflorescences from five sites in NS (Table 1).

As before, 0.50 g of seeds were cleaned from each inflorescence, but seeds from each plant were then divided into three separate Petri dishes. To each Petri dish, we added 40 ml of deionized water that was either fresh (the control), low salinity (4 ppt NaCl), or high salinity (18 ppt NaCl). Germination rates for each plant under each treatment were calculated in the same way as for the previously described genetic compatibility experiment.

#### 2.2.2 Growth in different salinities

We conducted an experiment to compare seedling performance under different salinity conditions to determine whether salinity (a) impedes the growth of *T*. × *glauca* seedlings more than *T. latifolia* seedlings, and (b) impedes the growth of ON *T. latifolia* more than NS *T. latifolia*. We grew seedlings from seeds that were produced by ten NS maternal parents of F_1_ hybrids, seven NS *T. latifolia* inflorescences, and eleven ON *T. latifolia* inflorescences (Table 1). We cleaned the seeds using the same methods described above, transferred them into Petri dishes containing deionized water, and then moved them to a greenhouse at Trent University in Peterborough, Ontario, with ambient light conditions and 18–20°C temperatures. After seven days, we transferred 40 seedlings from each Petri dish into 200‐cell plug trays filled with a premoistened germination‐grade soil (Sunshine professional growing soil #3 [Sun Gro Horticulture, Brantford, Ontario, Canada]). Plug trays were placed in flats that were approximately half‐filled with water and covered with clear plastic domes to maintain humidity until seedlings emerged from the soil and were ~10 cm tall. The water levels were maintained within the flats, and after five weeks we added 200 ml of 0.2% water‐soluble 20–20–20 N‐P‐K general purpose fertilizer (Peters Professional^®^, Scotts, Marysville, USA) weekly to each flat. After eight weeks, we transferred the plants into 10 cm pots filled with Sunshine Mix #1 Professional Growing Soil (Sungro, Agawam USA). We placed the pots in flats filled half‐way with water and added 400 ml of a 0.4% fertilizer solution weekly to the flats until the experiment began. At eleven weeks, plants were removed from their pots and rhizomes were trimmed to 12 cm. We transferred the trimmed plants into individual 8″ pots filled with Sunshine Mix #15, Professional Growing Soil (Sungro, Agawam USA), and placed each pot in a 5 L bucket filled with tap water. Water levels within buckets were checked daily and maintained at constant levels throughout the experiment. Each pot was randomly assigned to one of four treatments: 0, 4, 8, or 16 ppt NaCl. This left us with 161 plants that were divided among each of the four salinity treatments (11–16 plants per treatment × taxon combination).

Immediately prior to starting the experiment, offshoots within pots were labeled as ramets of the main plant to allow for later discrimination between pre‐existing and new ramets. At that time there were no differences in height between the *Typha* taxa (one‐way analysis of variance in longest leaf length among *Typha* taxa: *F*_2,160_ = 1.484, *p* > .05). Starting on July 1, 2018, we began weekly additions of 2 L of either tap water (control) or saline water at a concentration of 3 ppt; for plants receiving the latter, we continued to add solutions weekly until the water surrounding each pot had reached a concentration of either 4 (low saline), 8 (moderate saline), or 16 (high saline) ppt; these gradual additions were to allow the seedlings to acclimate to the salt treatments in order to reduce shock to the seedlings. We measured the salinity (direct concentration of Na ^+^ and Cl ^‐^ ions) within each bucket twice a week using a pocket salinity probe (Oakton PCTS Testr™ 50 Waterproof Pocket pH/Cond/TDS/Salinity Tester, Premium 50 Series), and by July 6, 12, and 26 the water in each pot had reached low, moderate, or high salinity, respectively. We continued to measure salinity twice weekly, and if any concentrations were ±1 ppt from their intended treatment levels we removed the water from the relevant bucket and replaced it with new solutions that had the correct salinity concentrations. We also added 50 ml of a 4% nutrient solution onto the soil surface of each pot once a week, along with 250 ml of the relevant NaCl treatment solutions.

After seedlings had been exposed to their target concentrations for a minimum of one week (i.e., one week after the buckets with high salinity had reached their target concentrations), we used a portable photosynthesis system (LI‐6400XT, Li‐Cor Inc., Lincoln, NE, USA) to measure three physiological responses to salinity: photosynthetic rate, stomatal conductance, and water‐use efficiency (ratio of the net photosynthetic rate per transpiration rate). These measurements were taken on 135 randomly selected individuals across the *Typha* taxa and different salinity treatments (5–7 individuals per treatment × taxon combination) between 9 a.m. and noon on August 1, 2, and 3, 2018. Light response curves on four randomly selected plants indicated a saturating response to light at ~800 μmol m^−2^ s^−1^. We therefore set the amount of photosynthetically active radiation of the portable photosynthesis system to 800 μmol m^−2^ s^−1^. We set CO_2_ concentration to 405 cm^3^/m^3^, leaf temperature to 25.0°C, and atmospheric flow rate to 0.5 dm^3^/min; all of these values were close to ambient levels. Readings were taken under ambient humidity. The portions of the leaves that were inside the photosynthesis system chamber where gas exchange measurements took place were then harvested and scanned to calculate leaf area using ImageJ (Rasband, 1997‐2018). Measured photosynthetic rates, stomatal conductances, and calculated water‐use efficiency were then adjusted based on the fraction of the leaf inside the chamber.

On the last day of the experiment, August 21, 2018 (day 52), we recorded survivorship of the main shoot and counted the number of new surviving ramets; these did not include ramets which predated the start of the experiment. Plants were considered alive when at least the 3 youngest leaves and half of the total leaves on the plant were living. We removed any leaves that had no green tissue and harvested the remaining aboveground shoots and leaves. We measured the length of the longest living leaf of each main shoot and then weighed the harvested material for each plant. Aboveground harvested matter was then dried in paper bags at 80°C in a 1.2 m × 1.5 m × 0.9 m drying oven (Binder BD 720, Binder GmbH, Tuttlingen, Germany), and re‐weighed after 72 hr.

### 2.3 Statistical analysis

#### 2.3.1 Germination in different salinities

Differences in germination success in fresh versus salt water between NS F_1_ hybrids and NS *T. latifolia,* and between NS *T. latifolia* and ON *T. latifolia,* were evaluated using generalized linear mixed‐effects models (GLMEs), with either taxon (for the NS *T. × glauca* vs. *T. latifolia* experiment) or province of origin (for the NS vs. ON *T. latifolia* experiment) and salinity treatment (0, 4, or 18 ppt) as crossed fixed effects predictors of the proportion of seeds that germinated. Because we included several levels of the salinity treatment in our experiment, this factor was specified as an ordinal independent variable and its effects were evaluated by testing for linear and nonlinear associations with response variables. Inflorescence nested within maternal site of origin was included as a random grouping variable. Models were calculated using a binomial error distribution (logit link) to meet model assumptions. Model parameters were calculated using the glmer function of the lme4 (v. 1.1‐21; (Bates et al., 2015) Bates et al., 2015) package in R (v. 3.6.1; (R Core Team, 2019).

### 2.4 Growth in different salinities

To assess the salinity tolerance of seedlings, we used separate models for each morphological and physiological response variable. For these tests, *Typha* group (NS *T. latifolia*, ON *T. latifolia*, or NS *T. × glauca*) and salinity treatment (0, 4, 8, or 16 ppt) and the interaction between *Typha* group and the salinity treatment were specified as fixed, independent variables. Salinity was specified as an ordinal independent variable. Maternal site of origin was included as a random grouping variable for tests involving morphological response variables. However, including site as a random effect in tests involving physiological response variables resulted in singular fits. For the physiological response variables, we report results from linear models.

Morphological response variables (survivorship and the number of surviving ramets) were modeled using GLMEs with the glmer function. For the test of survivorship, we specified binomial errors (logit link). For the test of the number of surviving ramets, we used a Poisson error term (log link) and the starting number of ramets was included as an offset. For the analyses of total aboveground biomass and longest leaf length we used linear mixed‐effects (LME) models using the lmer function of the lme4 package in R. To meet model assumptions, aboveground biomass was log‐transformed. The number of ramets at the start of the experiment was included as a covariate in the analysis of total aboveground biomass, and the longest leaf length at the start of the experiment was included as a covariate for the analysis of longest leaf length.

For each of the physiological response variables (photosynthetic rate, stomatal conductance, water‐use efficiency), we used linear models using the lm function in R. Stomatal conductance and water‐use efficiency were both log‐transformed to meet model assumptions. Water‐use efficiency required no transformation to meet model assumptions. For all models, including those described above, significance of fixed effects for models with a significant interactions term were calculated using type III sums of squares using the analysis of variance (ANOVA) function in the car package (v. 3.0‐3, Fox & Weisberg, 2019). For models with no significant interaction term we specified type II sums of squares. Pairwise comparison (Tukey) tests of the least‐square means were conducted using the lsmeans function in the lsmeans package (v. 2.30‐0, Lenth et al., 2019).

## 3 RESULTS

### 3.1 Genetic compatibility

Average seed set for the controlled‐cross NS F1 hybrids was 1965.58 ± 260.90 *SE* seeds per gram of fruit and mean percent seed germination was 94.20% ± 1.80 *SE*.

### 3.2 Germination in different salinities

**FIGURE 1:**
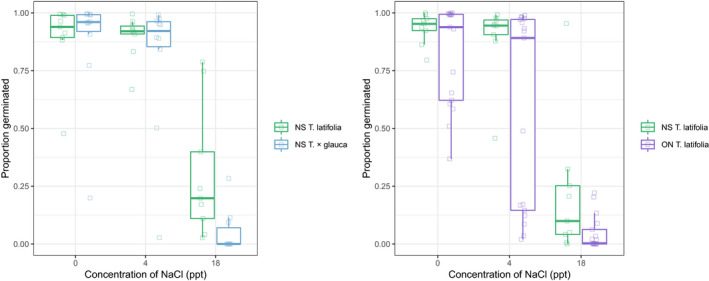
Variation in the proportion of cattail seeds that germinated under salinities of 0, 4 and 18 ppt. Shown are the results of an experiment comparing seed germination by F1 hybrid *Typha × glauca* and *T. latifiolia* plants from NS (A) and a separate experiment comparing seed germination by *T. latifolia* plants from NS and ON (b). Each box indicates the median (central line in each box), the upper and lower quartiles of the proportion of seeds that germinated for each maternal seed family × treatment level combination

High‐salinity conditions suppressed germination of NS *T. × glauca* more than NS *T. latifolia*, but these taxa had similar germination under freshwater or low‐salinity conditions, yielding a significant interaction between salinity treatment and *Typha* group (Table 2A, Figure 1a). The corresponding post hoc test revealed that the proportion of *T*. × *glauca* seeds that germinated was significantly lower than for *T. latifolia* under high‐salinity conditions (pairwise comparison test: *z*‐ratio = −3.092, *p* < .05), but not in the control (*z*‐ratio = −0.378, *p* > .95) or low salt treatments (*z*‐ratio = 1.085, *p* > .85). Moreover, seeds from seven out of the ten sampled *T. × glauca* plants had 0% germination under high salinity, with germination rates for the remaining three plants ranging from 9%–28% (Table S1). In comparison, germination rates of *T. latifolia* seeds under high salinity ranged from 4%–78% (Table S1). In the high‐salinity treatment, mean percent germination was 4.92% (standard error: ±2.8) for NS *T*. × *glauca*, and 30.2% (standard error: ±9.00) for NS *T. latifolia* (Figure 1a, Table S1).

**TABLE 2:** Variation in the germination, growth and physiological responses of *Typha* groups (NS *T. × glauca,* NS *T. latifolia,* ON *T. latifolia*) to different levels of salinity under common environmental conditions

	Salinity	Group	Salinity × Group
(A) Germination			
NS *T. × glauca* vs. NS *T. latifolia*	χ^2^ (2) = 3.41 × 10^3^***	χ^2^ (1) = 1.62^NS^	χ^2^ (2) = 4.26 × 10^2^***
NS *T. latifolia* vs. ON *T. latifolia*	χ^2^ (2) = 2.85 × 10^3^***	χ^2^ (1) = 1.86^NS^	χ^2^ (2) = 3.58 × 10^2^***
(B) Growth			
Survivorship	χ^2^ (3) = 14.8**;	χ^2^ (2) = 8.53*; 2 *df*	χ^2^ (6) = 6.00^NS^
Ramet production	χ^2^ (3) = 20.3***	χ^2^ (2) = 20.1***	χ^2^ (6) = 16.2*
Aboveground biomass	χ^2^ (3) = 88.6***	χ^2^ (2) = 0.94^NS^	χ^2^ (6) = 7.60^NS^
Leaf length	χ^2^ (3) = 1.03 × 10^2^***	χ^2^ (2) = 1.45^NS^	χ^2^ (6) = 7.35^NS^
(C) Physiology			
Photosynthesis	*F*_3,123_ = 21.7***	*F*_2,123_ = 2.13^NS^	*F*_6,123_ = 1.10^NS^
Conductivity	*F*_3,123_ = 11.4***	*F*_2,123_ = 9.00***	*F*_6,123_ = 2.45*
Water‐use efficiency	*F*_3,123_ = 13.0***	*F*_2,123_ = 0.96^NS^	*F*_6,123_ = 0.70^NS^

Note

Shown are test‐statistic values and their associated degrees of freedom. For A and B (Germination and Growth) test statistics are analysis of deviance Wald Chi‐square values (with degrees of freedom in parentheses). For C (Physiology) test statistics are analysis of variance *F*‐test values (with numerator and denominator degrees of freedom indicated using subscripts).

* *p* < .05; ** *p* < .01; *** *p* < .001.

There was a significant interaction between salinity treatment and province of origin (Table 2A, Figure 1b) that appeared to emanate from similar germination rates in freshwater, but lower germination rates under high salinity, for ON*T. latifolia* compared to NS *T. latifolia*. However, the post hoc test did not provide further support for this interpretation (pairwise comparison test NS versus ON *T. latifolia* under high salinity: *z*‐ratio = 2.32, *p* > .15). In the high‐salinity treatment, mean percent germination was 21.48% (standard error: ±9.43) for NS *T. latifolia*, and 4.6% (standard error: ±1.72) for ON *T. latifolia* (Figure 1b).

### 3.3 Growth in different salinities

**FIGURE 2:**
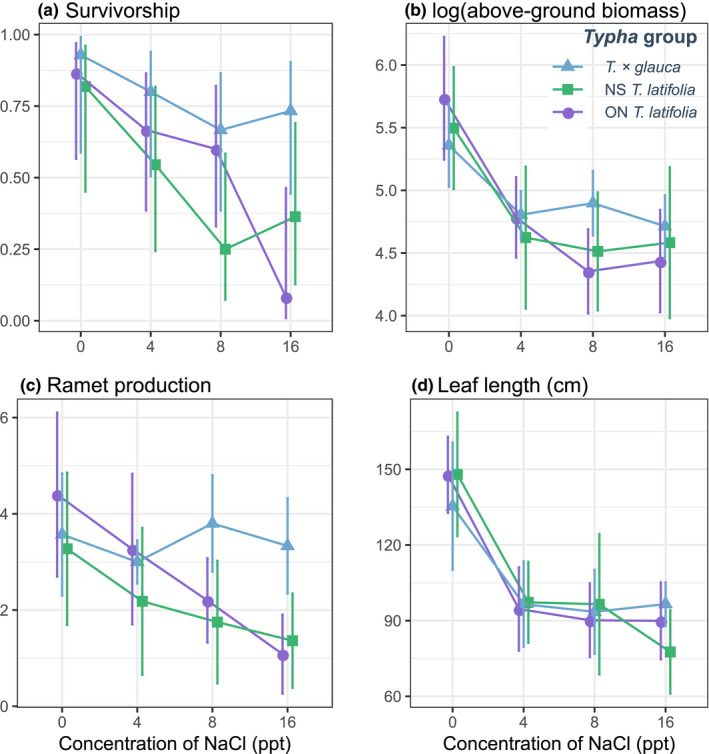
Interaction diagrams indicating the mean and standard error survivorship (a), aboveground biomass (b), ramet production (c), and leaf length (d) of cattail plants grown under salinities of 0, 4, 8, and 16 ppt. Each line represents the response of F1 hybrid *Typha × glauca* and *T. latifiolia* plants from NS and of *T. latifolia* plants from ON to each level of the salinity treatment. Data are plotted on a response scale

The effect of salinity on seedling survival and ramet production differed among *Typha* taxa. On average, survivorship for ON *T. latifolia* and NS *T. latifolia* were lower than for NS *T*. × *glauca*, and this difference was most apparent under high salinities (Table 2B, Figure 2a). The corresponding post hoc test revealed that survivorship of ON *T. latifolia* was significantly lower under high salinity versus control conditions (pairwise comparison test: *z*‐ratio = 3.31, *p* < .05), whereas there was no difference in survivorship of either *T*. × *glauca* or NS *T. latifolia* in high‐salinity conditions compared to control conditions (control‐high *z*‐ratio = 1.33, *p* > .95 and *z*‐ratio = 2.02, *p* > .50, respectively). In spite of these contrasting responses to salinity between taxa, there was no significant interaction between salinity and *Typha* group. Moreover, although there was a significant effect of salinity treatment on survivorship (Table 2), there were no significant linear or higher‐order associations between levels of the salinity treatment and survivorship (linear parameter estimate = −1.22 ± 0.83 *SE* *z* = −1.48, *p* > .10).

Salinity significantly reduced the production of new ramets for ON *T. latifolia* and NS *T. latifolia*, but not for NS *T*. × *glauca*, yielding a significant interaction term for *Typha* group and salinity (Table 2B, Figure 2b). In general, there was a negative association between salinity level and ramet production (first‐order linear parameter estimate for salinity = −0.44 ± 0.14 *SE*, *z* = −3.02, *p* < .01). However, these effects were stronger for *Typha latifolia* from ON and NS than they were for *T*. × *glauca* from NS. The corresponding post hoc test supported this inference and indicated that under high‐salinity conditions NS *T*. × *glauca* produced significantly more ramets than NS *T. latifolia* (pairwise comparison: *z*‐ratio = 4.11, *p* < .01). Additionally, both ON *T. latifolia* and NS *T. latifolia* had significantly more ramets under control versus high‐salinity conditions (*z*‐ratio = 5.65, *p* < .001 and *z*‐ratio = 3.93, *p* < .01, respectively), whereas ramet production by NS *T*. × *glauca* was not significantly different between these treatment levels (*z*‐ratio = 3.00, *p* > .10).

There was no evidence that *Typha* taxa and salinity interacted with one another to influence aboveground biomass or leaf length (Table 2B, Figure 2c,d). Overall, salinity significantly reduced aboveground biomass and leaf length for the three *Typha* taxa (first‐order linear parameter estimates: biomass = −4.82 ± 0.13 *SE*, *z* = −3.65, *p* < .001; leaf length = −27.2 ± 6.89 SE, *z* = −3.95, *p* < .001). The post hoc test revealed that aboveground biomass of both NS and ON*T. latifolia* was significantly higher under control conditions versus low, moderate, and high‐salinity conditions (pairwise contrasts for NS *T. latifolia*: control‐low *t*‐ratio = 3.58, *p* < .05, control‐moderate *t*‐ratio = 4.21, *p* < .01, and control‐high *t*‐ratio = 4.45, *p* < .01; ON *T. latifolia*: control‐low *t*‐ratio = 4.45, *p* < .01, control‐moderate *t*‐ratio = 6.22, *p* < .001, and control‐high *t*‐ratio = 5.505, *p* < .001), and aboveground biomass production by *T*. × *glauca* was significantly higher in the control treatment compared to the high salt treatment (*t*‐ratio = 3.70, *p* < .05). Post hoc tests for leaf length similarly indicated declining plant size with salinity. The length of the longest leaf was significantly higher under control conditions versus low, moderate, and high‐salinity conditions for all *Typha* groups (all pairwise contrast *t*‐ratios > 3.5, *p* < .05).

**FIGURE 3:**
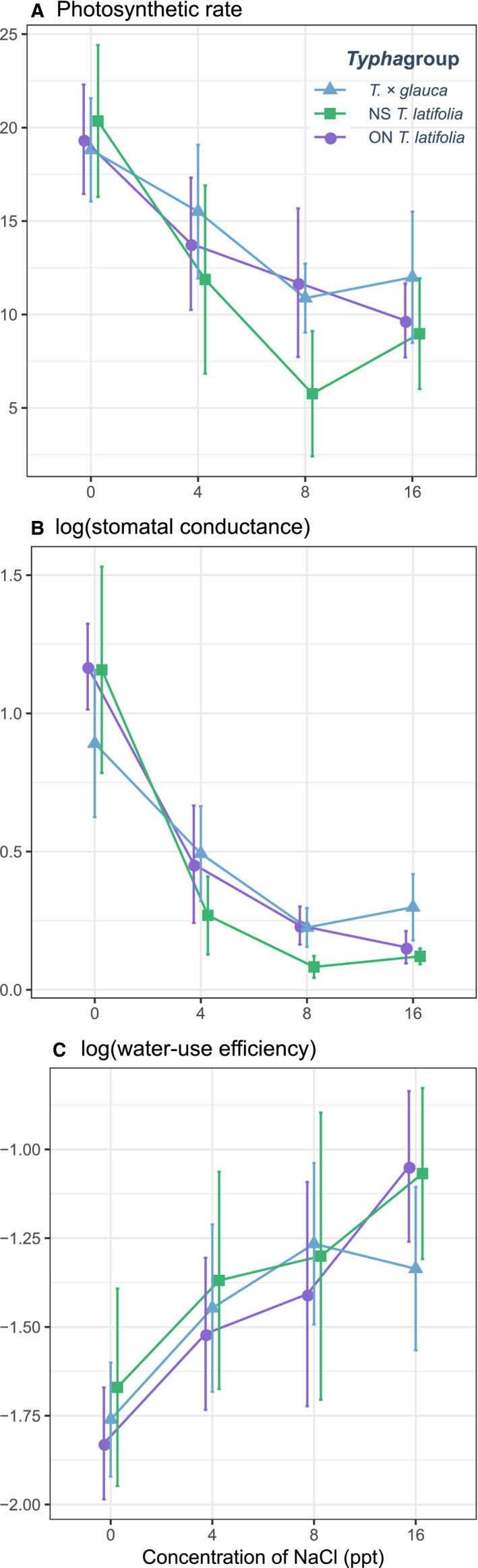
Interaction diagrams indicating the mean and standard error photosynthetic rates (a), stomatal conductances (b), and water‐use efficiencies (c) of cattail plants grown under salinities of 0, 4, 8, and 16 ppt. Each line represents the response of F1 hybrid *Typha × glauca* and *T. latifiolia* plants from NS and of *T. latifolia* plants from ON to each level of the salinity treatment. Data are plotted on a response scale

There was also no evidence that *Typha* group and salinity interacted with one another to influence photosynthetic rate or water‐use efficiency (Table 2C). Overall, higher salinity was associated with significantly reduced photosynthetic rates and stomatal conductances, and increased water‐use efficiency, for the three *Typha* groups compared to control levels (Table 2, Figure 3). For all *Typha* groups photosynthetic rates and stomatal conductances declined significantly with salinity (first‐order linear parameter estimates: photosynthesis rate = −5.61 ± 1.53 *SE*, *z* = −3.65, *p* < .001; conductivity = −0.97 ± 0.18, *z* = −5.33, *p* < .001) and water‐use efficiency increased with salinity (first‐order linear parameter estimate for water‐use efficiency = 0.32 ± 0.11 *SE*, *z* = 2.93, *p* < .01). However, stomatal conductance was also associated with salinity in a nonlinear manner (second‐order quadratic parameter estimate = 0.44 ± 0.18 *SE*, *z* = −2.14, *p* < .05) indicating a decelerating association between salinity and stomatal conductance that was more apparent for NS *T. glauca* and *T. latifolia* than for ON *T. latifolia* (Figure 3b). These different higher‐order associations between salinity and conductance appeared to drive a significant interaction between salinity levels and *Typha* groups (Table 2C). However, in spite of these indicated differences in response among *Typha* groups, stomatal conductances declined strongly with salinity for all taxa.

## 4 DISCUSSION

In this study, we used a combination of field and greenhouse experiments to investigate whether genetic incompatibilities or environmental conditions can help to explain why the hybrid *T*. × *glauca* is relatively rare in NS coastal wetlands, despite its success as a dominant invasive species in regions surrounding the Laurentian Great Lakes and St. Lawrence Seaway. We found no evidence to support our hypothesis that genetic incompatibility between progenitor species in NS leads to inviable hybrids, nor did we find support for the hypothesis that saline conditions reduce growth of hybrid plants. However, our data did support the hypothesis that conditions of high salinity reduced germination rates of hybrid seeds more than germination rates of NS *T. latifolia*. Our data are also consistent with the hypothesis that NS *T. latifolia* is locally adapted to saline conditions, which raises the possibility that competition is limiting the spread of hybrids in NS. Below we will discuss these findings and consider how our study can help us to understand why *T. × glauca* is a dominant invader at only some contact zones.

### 4.1 Genetic compatibility of progenitor species

The average seed set for the NS hybrids of 1965.58 seeds per gram of fruit was comparable to the value of 2709.40 seeds per gram of fruit found in ONF1 hybrids in a previous study (Pieper et al., 2017). Additionally, the germination rate of 94.20% was higher than the value of 65.20% previously recorded in ON F1 hybrids (Pieper et al., 2017). We therefore conclude that *T. latifolia* and *T. angustifolia* in NS are genetically compatible and can form large numbers of viable F1 hybrid seeds; as a result, we found no support for the hypothesis that genetic incompatibility can explain the relative scarcity of *T*. × *glauca* in NS. This is consistent with a number of other studies which concluded that genetic incompatibility cannot explain variations in hybrid frequencies across multiple contact zones (e.g., Campbell et al., 2008; Howard, 2001; Li & Maki, 2015; Watano et al., 2004). For example, instead of genetic incompatibility, pollinator flower choice in *Ipomopsis aggregata* and *I. tenuituba* seems to be an important determinant of hybrid frequencies across contact zones (Aldridge & Campbell, 2009), and landscape modifications including drainage ditches influence hybrid frequencies between *Rorippa amphibia* and *R. palustris* (Bleeker & Hurka, 2001).

### 4.2 High salinity limits hybrid seed germination

In the absence of genetic barriers, frequencies of interspecific hybrids may be limited by reduced fitness in certain environmental conditions. Although the novel gene combinations and relatively high levels of heterozygosity that are typically found in hybrids can facilitate their adaptation to different environments (e.g., Ellstrand & Schierenbeck, 2000; Pfennig et al., 2016; Wolfe et al., 2007), hybrids can also experience reduced fitness following a loss of co‐adapted gene complexes or locally adapted alleles (Palacio‐Lopez et al., 2017; Waser & Price, 1989). This may explain why *T*. × *glauca* in NS has significantly reduced germination compared to *T. latifolia* under conditions of high salinity. A growing number of studies have determined that local environmental conditions such as climate, temperature, salinity, and habitat can be important determinants of hybrid frequencies (Carson et al., 2012; Culumber et al., 2012; De La Torre et al., 2014; Walsh et al., 2016). Our data add to this literature because we found support for the hypothesis that environmental salinity impedes the germination of *T. *× *glauca*; therefore, high salinity could help to explain the relatively low frequencies of hybrids in the coastal wetlands of NS.

Other studies have similarly found that salinity can affect seed germination rates in both hybrids and their progenitor species. Hybrids of the coastal perennials *Carpobrotus edulis* and *C. chilensis,* which can be abundant in tidal marshes, had higher germination rates than those of either parent species under low to moderate levels of salinity (Weber & D'Antonio, 1999), although a later study also found that there is more variation in germination requirements and salinity tolerance in the hybrids compared to the parental species *C. edulis* (Podda et al., 2018). Salt tolerance in seeds is an important trait for plants growing in coastal habitats, where higher salinity levels reduce the germination rates of many species (Baskin & Baskin, 2014). Although *Typha* spp. reproduce both clonally and sexually, clonal reproduction is unlikely to compensate for reduced germination rates because approximately half of the recruitment within stands of both *T. latifolia* and *T*. × *glauca* is from seeds (Pieper et al., 2020); therefore, a reduced germination rate under highly saline conditions could hinder the establishment and expansion of *T*. × *glauca* in coastal wetlands.

### 4.3 High salinity does not limit hybrid growth

Although germination of *Typha × glauca* seeds was suppressed under high salinity, the performance of hybrid plants was comparable or superior to *T. latifolia* under highly saline conditions. There were few substantial differences in biomass or survival between NS *T. latifolia* and *T*. × *glauca* across salinity levels. However, *T. latifolia* growing in high salinity produced fewer ramets than when growing in freshwater and low salinity. Hybrids, on the other hand, produced similar numbers of ramets regardless of salinity, and produced significantly more ramets than *T. latifolia* under high salinity. Other studies have found relatively high levels of salt tolerance in hybrids, for example, the hybrid sunflower *Helianthus paradoxus* had higher survival and biomass than either parent species under highly saline conditions, and is considered to be transgressive for salt tolerance relative to its progenitors (Welch & Rieseberg, 2002) (Karrenberg et al., 2006). In another example, the hybrid of *Spartina densiflora* and *S. foliosa* appears better able than its parent species to deal with the stressors of flooding and salinity associated with sea level rise (Gallego‐Tévar et al., 2020). The relatively high production of ramets by *T. × glauca* provides an important mechanism for clonal expansion within a coastal site. As noted above, clonal growth is responsible, on average, for roughly half of *Typha* recruitment in eastern Canada (Pieper et al., 2020), and therefore plants that can expand clonally could have an advantage. However, clonal expansion is reliant upon initial colonization of a site, and may also rely on repeated recolonization of a site following disturbances such as water level fluctuations; both colonization and recolonization should be greatly hindered by low germination rates by hybrids in saline wetlands. We therefore suggest that although established *T*. × *glauca* may be able to outcompete *T. latifolia* in highly saline coastal wetlands, inhibited germination is one factor limiting the spread of hybrids in these habitats. Collectively these findings also highlight the importance of considering different life stages when assessing environmental adaptations. Beare and Zedler (1987) similarly found life history stage to be important: while seeds and seedlings of the congeneric species *T. domingensis* were sensitive to salt, older plants were much more salt tolerant.

Plants have multiple mechanisms for dealing with ionic stress in saline environments (reviewed in Flowers & Colmer, 2008, 2015; Hasegawa et al., 2000). These mechanisms can be assessed in a number of different ways including photosynthetic rate and stomatal conductance which may decrease under salt stress to avoid excessive water loss from transpiration (Welch & Rieseberg, 2002; Yeo, 1998). Our results were consistent with each of these expectations: increased levels of salinity led to reductions in photosynthetic rate and stomatal conductance, with the latter leading to increases in water‐use efficiency. These responses are common among plants that avoid salt ion uptake (Chaves et al., 2009; Munns et al., 2006), for example, the coastal‐marsh halophyte species *Juncus roemerianus* achieved salt‐avoidance through decreased stomatal conductance (Touchette et al., 2009). However, the responses we observed in this study were similar across *Typha* groups (*T*. × *glauca*, NS *T. latifolia*, ON *T. latifolia*), indicating that physiological mechanisms associated with photosynthesis and water‐use efficiency are unlikely to explain regional variation in patterns of cattail hybrid formation.

### 4.4 Salinity tolerance in *T. latifolia*

Our germination and growth experiments also tested the hypothesis of local adaptation to salt in NS *T. latiolia* by comparing germination and growth between *T. latifolia* from Ontario, where salinity is overall low, and *T. latifolia* from NS where wetlands often have high salinity. We found that under conditions of high salinity, germination rates were lower in ON *T. latifolia* compared to NS *T. latifolia*. These findings suggest that NS *T. latifolia* is better adapted than ON *T. latifolia* to saline environments, which in turn may help *T. latifolia* to outcompete *T*. × *glauca* in saline environments. It is not uncommon for conspecific plants to exhibit local adaptation to contrasting environmental conditions in different parts of their range, for example, *Polypogon monspeliensis* (Atia et al., 2011), *Rouya polygama* (Santo et al., 2014)*, Crithmum maritimum* (Marchioni‐Ortu & Bocchieri, 1984), and *Medicago trunculata* (Cordeiro et al., 2014) have all shown evidence of variable seed germination rates under different levels of salinity, depending on the provenance of the seeds. Similarly, plants in the congeneric species *Typha domingensis* showed different levels of tolerance to salinity depending on their provenance: seeds collected from plants growing in a more saline area were more salt tolerant than those from a less saline area (Beare & Zedler, 1987).

## 5 CONCLUSIONS

The field and greenhouse experiments conducted in this study collectively suggest that the low frequencies of *T*. × *glauca* in NS compared to ON are at least partly attributable to reduced seed germination and hence low recruitment in conditions of high salinity. Our data also suggest that NS *T. latifolia* is better adapted to high salinity than ON*T. latifolia,* and may therefore be particularly competitive in coastal wetlands. Because *T*. × *glauca* has one parent that performs well in saline conditions, the low germination rates following hybridization could be explained by either a disrupted co‐adapted gene complex, or the loss of alleles that confer adaptation to highly saline conditions, and which may be lacking in the maternal parent *T. angustifolia*. Perhaps surprisingly, *Typha angustifolia* has been reported as having greater salinity tolerance than *T. latifolia* (McMillan, 1959), although the performance of *T. angustifolia* in saline conditions can be strongly influenced by nutrient availability (Smith et al., 2015). Furthermore, previous investigations of *T. angustifolia* salinity tolerance have been based on plant growth and not germination rates. Future studies could investigate whether NS *T. angustifolia* also shows low germination rates in highly saline conditions, because this might help to explain both the reduced germination in *T*. × *glauca* relative to *T. latfolia,* and the scarcity of *T. angustifolia* in the coastal wetlands of eastern Canada. More broadly, our study has added to the growing body of literature (e.g., Carson et al., 2012; Tarroso et al., 2014) which identifies local habitat and adaptation as playing important roles in the distributions and characteristics of hybrid zones.

## CONFLICT OF INTEREST

The authors have no conflicts of interest to declare.

## AUTHOR CONTRIBUTION

**Kathryn Tisshaw:** Conceptualization (equal); Formal analysis (lead); Investigation (lead); Methodology (lead). **Joanna Freeland:**Conceptualization (equal); Supervision (equal); Funding acquisition (equal); Writing review editing (lead); Methodology (equal). **Marcel Dorken:** Conceptualization (equal); Supervision (equal); Funding acquisition (equal); Methodology (equal); Formal analysis (equal); Review editing (equal).

## Supporting information

Appendix S1

## Data Availability

The data supporting the results in this study are archived in Dryad, https://doi.org/10.5061/dryad.3tx95x6dh.
