# Supplementary material for: Salinity, not genetic incompatibilities, limits the establishment of the invasive hybrid cattail Typha × glauca in coastal wetlands
Source: Ecol Evol. 2020 Oct 1;10(21):12091–103. doi: 10.1002/ece3.6831 (PMC7663983; doi:10.1002/ece3.6831)
Supplement: Supplementary file 1 — Appendix S1 [file ECE3-10-12091-s001.docx]

**Appendix**

Table S1. Proportion of seeds of *T. × glauca* and *T. latifolia* that germinated in each of the three salinity treatments with means and standard errors (SE).

|  | Proportion of germinated seeds | | |
| --- | --- | --- | --- |
|  | Control | Low salinity | High salinity |
| ***T. × glauca*** |  |  |  |
| Blueberry Run Trail | 0.993 | 0.991 | 0 |
| Blueberry Run Trail | 0.994 | 0.965 | 0.284 |
| Brooklyn Street | 0.199 | 0.028 | 0 |
| Brooklyn Street | 0.959 | 0.502 | 0 |
| Exit 7 | 0.773 | 0.953 | 0 |
| Exit 7 | 0.997 | 0.974 | 0.094 |
| Exit 9 | 0.956 | 0.890 | 0.114 |
| Exit 9 | 0.962 | 0.842 | 0 |
| Irishman’s Road | 0.907 | 0.894 | 0 |
| Irishman’s Road | 0.990 | 0.949 | 0 |
| **Mean** | **0.873** | **0.799** | **0.0492** |
| SE | 0.074 | 0.092 | 0.028 |
| ***T. latifolia*** |  |  |  |
| Irishman’s Road | 0.995 | 0.909 | 0.748 |
| Irishman’s Road | 0.989 | 0.996 | 0.788 |
| Irishman’s Road | 0.964 | 0.943 | 0.041 |
| Irishman’s Road | 0.882 | 0.833 | 0.110 |
| Lawrencetown Coastal Heritage Park | 0.478 | 0.669 | 0.399 |
| Miner’s Marsh | 0.913 | 0.921 | 0.198 |
| Miner’s Marsh | 0.993 | 0.962 | 0.027 |
| Miner’s Marsh | 0.893 | 0.926 | 0.240 |
| Miner’s Marsh | 0.939 | 0.917 | 0.171 |
| **Mean** | **0.894** | **0.897** | **0.302** |
| SE | 0.051 | 0.030 | 0.090 |

Table S2. Proportion of seeds of ON*T. latifolia* and NS *T. latifolia* that germinated in each of the three salinity treatments with means and standard errors (SE).

|  | Proportion of germinated seeds | | |
| --- | --- | --- | --- |
|  | Control | Low salinity | High salinity |
| **NS *T. latifolia*** |  |  |  |
| Irishman’s Road | 0.938 | 0.992 | 0.324 |
| Irishman’s Road | 1 | 0.977 | 0.954 |
| Irishman’s Road | 0.924 | 0.945 | 0.252 |
| Irishman’s Road | 0.862 | 0.878 | 0.099 |
| Lawrencetown Coastal Heritage Park | 0.994 | 0.944 | 0.006 |
| Miner’s Marsh | 0.953 | 0.906 | 0.206 |
| Miner’s Marsh | 0.965 | 0.969 | 0.041 |
| Miner’s Marsh | 0.796 | 0.458 | 0.05 |
| Miner’s Marsh | 0.975 | 0.949 | 0 |
| **Mean** | **0.934** | **0.891** | **0.215** |
| SE | 0.0209 | 0.0523 | 0.0943 |
| **ON*T. latifolia*** |  |  |  |
| Cottage Road | 0.994 | 0.958 | 0.034 |
| Cottage Road | 0.995 | 0.932 | 0.221 |
| Cottage Road | 1 | 0.984 | 0.134 |
| Cottage Road | 0.999 | 0.990 | 0 |
| Elm Tree Road | 0.992 | 0.981 | 0.063 |
| Elm Tree Road | 0.744 | 0.02 | 0 |
| Elm Tree Road | 0.622 | 0.086 | 0.003 |
| Elm Tree Road | 0.606 | 0.167 | 0.003 |
| Heron Landing Golf Course | 0.992 | 0.971 | 0.089 |
| Heron Landing Golf Course | 0.991 | 0.921 | 0 |
| Heron Landing Golf Course | 0.584 | 0.036 | 0 |
| Sand Bar Road | 0.996 | 0.975 | 0.021 |
| Sand Bar Road | 0.938 | 0.891 | 0.203 |
| Sand Bar Road | 0.930 | 0.489 | 0.019 |
| University and 4^th^ Line | 0.654 | 0.146 | 0 |
| University and 4^th^ Line | 0.368 | 0.171 | 0 |
| University and 4^th^ Line | 0.510 | 0.123 | 0 |
| **Mean** | **0.819** | **0.579** | **0.046** |
| SE | 0.0508 | 0.0998 | 0.0172 |
